# Literary Notes

**Published:** 1906-04

**Authors:** 


### LITERARY NOTES.

The Kansas City Medical Record gets itself into a new spring costume with its March issue. Its cover, in speckled bluish-gray and red is a dream, while the new arrangement of its pages gives it a look of quality that indicates a prosperity we are glad to witness.

American Medicine announces that from and after April 1, 1906 it will appear as a monthly magazine. We regret to lose the weekly visits of this staunch defender of the medical faith, but, since it has become necessary to change, we offer the right hand of fellowship at the threshold as our monthly contemporary enters the temple.

The New York State Journal of Medicine, under the editorship of Dr. James P. Warbasse, has appeared in a fine new dress to celebrate the Centenary of the Medical Society of the State of New York, which organization the journal represents. The bright orange color of the cover is attractive but the contents is the main part of a medical magazine. The March number contains the addresses of the centennial meeting, as well as much other material of absorbing interest to every physician in the state. We are more than pleased with the way the new editor begins, and bespeak for him the support of his editorial colleagues throughout the land.

Messrs. P. Blakiston's Son and Company, Philadelphia, announce the publication of an English edition of Casper's Lehrbuch der Urologie, which has been prepared with the permission and collaboration of the author by Dr. Charles W. Bonney, of Philadelphia. The intristic excellence of this work and the great favor accorded it in the original, has led to the preparation of the forthcoming edition, which is now offered to English speaking practitioners and students of medicine and surgery as a modern, scientific and practical textbook on the diseases of the genitourinary organs.

---

THE NEW INTERNATIONAL ENCYCLOPEDIA, which is now regarded as the acme of reference works, is destined to have a tremendous influence in the educational affairs of the world. It is considered the greatest achievement in its line ever attempted in this country. Although only recently completed it is rapidly supplanting the old encyclopedias. Being strictly new and original it is more valuable than any revised or supplemented work can be. The New International is eminently practical, larger than any other reference work in the English language, and beautifully gotten up so far as maps, illustrations, and mechanical features are concerned. To say the least, it is of exceptional interest and is receiving wide recognition and commendation. It is the one schools and libraries are buying almost exclusively.

